# Nature-based agricultural solutions: Scaling perennial grains across Africa

**DOI:** 10.1016/j.envres.2017.08.011

**Published:** 2017-11

**Authors:** Brad G. Peter, Leah M. Mungai, Joseph P. Messina, Sieglinde S. Snapp

**Affiliations:** a Department of Geography, Environment, and Spatial Sciences, Michigan State University, East Lansing, MI, USA; b Department of Plant, Soil and Microbial Sciences, Michigan State University, East Lansing, MI, USA; c Center for Global Change and Earth Observations, Michigan State University, East Lansing, MI, USA

**Keywords:** Nature-based solutions, Crop suitability, Scaling, Perennial grains, Remote sensing

## Abstract

Modern plant breeding tends to focus on maximizing yield, with one of the most ubiquitous implementations being shorter-duration crop varieties. It is indisputable that these breeding efforts have resulted in greater yields in ideal circumstances; however, many farmed locations across Africa suffer from one or more conditions that limit the efficacy of modern short-duration hybrids. In view of global change and increased necessity for intensification, perennial grains and long-duration varieties offer a nature-based solution for improving farm productivity and smallholder livelihoods in suboptimal agricultural areas. Specific conditions where perennial grains should be considered include locations where biophysical and social constraints reduce agricultural system efficiency, and where conditions are optimal for crop growth. Using a time-series of remotely-sensed data, we locate the marginal agricultural lands of Africa, identifying suboptimal temperature and precipitation conditions for the dominant crop, i.e., maize, as well as optimal climate conditions for two perennial grains, pigeonpea and sorghum. We propose that perennial grains offer a lower impact, sustainable nature-based solution to this subset of climatic drivers of marginality. Using spatial analytic methods and satellite-derived climate information, we demonstrate the scalability of perennial pigeonpea and sorghum across Africa. As a nature-based solution, we argue that perennial grains offer smallholder farmers of marginal lands a sustainable solution for enhancing resilience and minimizing risk in confronting global change, while mitigating social and edaphic drivers of low and variable production.

## Highlights

• Mapping suitability and scaling potential of perennial crops across Africa.
• Targeting agricultural development through remote sensing-based classification.
• Locating areas for deployment of nature-based sustainable land management strategies.

## 1 Introduction

### 1.1 Adapting agriculture in view of a changing landscape

Globally, there is a call to increase food production through sustainable practices, to counter the effects of climate change, rising population, land-use change, and deterioration of natural resources (Postel, 2000, Godfray et al., 2010). Subsistence agriculture in Africa in particular is becoming increasingly stressed by population pressures, soil exhaustion, and climate change. Since the 1990's, the population of Africa has been growing at a rate faster than the global average (Reilly and Schimmelpfennig, 1999), contributing to the conversion of land for both agricultural production and urbanization. Climate variability, such as unpredictable timing and quantity of rainfall, affect smallholder farmer crop production and farm management practices (Nelson et al., 2009), particularly in marginal environments (Reilly and Schimmelpfennig, 1999). These changes, among others, have prompted increased research for agricultural intensification (Josephson et al., 2014, Ricker-Gilbert et al., 2014). Sub-Saharan African agricultural systems are among the most vulnerable systems facing these challenges (Challinor et al., 2007), and ongoing research that is both innovative and participatory will foster biodiversity and increased productivity (Snapp et al., 2010).

### 1.2 Sustainable land management and emergent nature-based solutions

Sustainable land management is at the foundation of societal goals to produce food, fuel, and fodder in an environmentally sound and supportable manner over the long-term. How to achieve sustainable intensified agriculture is contested, with advocates promoting such various approaches as organic agriculture, agroecology, and conservation farming (Petersen and Snapp, 2015). At the same time, there are a number of sustainability principles about which consensus is emerging that can be followed regardless of the type of agriculture deployed (Robertson et al., 2014). Notable among these is the case for perennial vegetation (Pimentel et al., 1986, Glover et al., 2010). This type of plant trait ensures greater capture and conservation of resources, as living plants are continuously present to photosynthesize and foster biological processes such as nutrient recycling (Jackson, 2002). Thus, a range of alternative management practices, including organic and conservation agriculture can potentially derive considerable benefit from integration of perennials. In contrast, agriculture as currently practiced involves crops with overwhelmingly annual growth habits. This leads to managed lands left bare or unproductive for much of the year, as most food crops are grown for a scant four to five months (Jackson, 2002). There are sustainable alternatives to such annual-intensive production, which include mixed cropping systems that incorporate perennial plants as companion species and the development and deployment of crop plants that have perennial properties. Sorghum and pigeonpea are two such crops with perennial growth habits that allow regrowth and multiple harvests through an agronomic practice called ratooning (Rogé et al., 2016), which involves cutting the main stem(s) after reproductive maturity.

Based on agroecology and conservation agriculture principles, nature-based solutions offer sustainable qualities, such as resilience to disturbance over variable time scales and minimal input requirements, as well as an aim toward general improved human well-being (European Commission, 2015). Nature-based solutions for sustainable urbanization emerged in the European Union as a research initiative with the explicit aim of addressing environmental, social, and economic challenges sustainably (European Commission, 2015). Similarly, smallholder agricultural systems will benefit from adoption of technologies that support sustainable principles and the complex system processes of nature. At the same time, the “net benefit of nature-based solutions depends on how much non-renewable energy can be replaced without decreasing total production of ecosystem services” (Maes and Jacobs, 2015: 3). Attention to resilience and resource amelioration is often overlooked in agricultural discourse in favor of the intensification of high-yield annuals, which dominates the development lexicon. In contrast to nature-based solutions that focus on economic solutions (e.g., fertilizer subsidy), nature-based solutions might be better expressed in the smallholder agricultural context through sustainable soil management strategies, field/crop organization, and local market development.

### 1.3 Perennial grains: a nature-based approach for Africa

Pigeonpea (*Cajanus cajan* (L.) Millspaugh) is a perennial leguminous shrub commonly cultivated and consumed by smallholder farmers in the tropical and often marginal environments of Africa (Kumar Rao and Dart, 1987). Pigeonpea is biologically suited to production as an intercrop, as a slow initial growth habit minimizes competition for resources with a primary crop (Sakala et al., 2000). Farmers typically grow pigeonpea as a mixed cropping system, producing two or more food products while simultaneously building soil fertility through biological processes (e.g., nitrogen fixation and carbon sequestration) (Fujita et al., 1992, Snapp et al., 2010). Sorghum (*Sorghum bicolor* (L.) Moench) is a grass with perennial properties commonly grown by smallholder farmers in semi-arid regions of Africa (Doggett, 1988, Paterson et al., 2009). The cultivation of sorghum occurs primarily in dryland areas and hot, semi-arid tropical environments (Dicko et al., 2006), though there are sorghum species that grow in wetter regions (Harlan and Stemler, 1976). Sorghum has deep and spread roots and a solid stem (Buchanan, 1885), and it is common to grow sorghum along field ridges, as this helps prevent soil erosion (Okigbo and Greenland, 1976). Pigeonpea and sorghum are generally drought-tolerant and resilient in dryland environments, and in perennial forms maintain these desirable traits (Parr et al., 1990).

A research gap that has not been addressed is the spatial assessment of suitability for diversification of nature-based solutions. In this paper, we consider the phenology of two perennial crops, pigeonpea and sorghum, both of which offer soil rehabilitation properties and are tolerant to marginal environments (Okigbo and Greenland, 1976, Snapp et al., 2010). The unique properties of these crops enhance sustainability of cropping, however they face biophysical constraints and require an appropriate socio-economic context to achieve successful adoption. Our overall objective is to identify appropriate integration properties and delineate the climate niche for deployment of these sustainable perennials within existing maize-based smallholder farms. From an agroclimatology perspective, we explore areas suitable for the cultivation of perennial sorghum and pigeonpea, and highlight the geographic potential for perennial grains to scale across Africa. Remotely-sensed imagery such as those presented here allow for global observation of the environmental and biophysical factors that influence perennial development and adaptation and their spatial organization. Identifying biogeographic conditions in which sorghum and pigeonpea can prosper is crucial for effective integration and scaling of these perennial grains (Wood et al., 1999).

This work builds on other global and continental suitability maps (e.g., the Global Agro-Ecological Zones (GAEZ) database (IIASA/FAO, 2012)) by highlighting the intersection between marginal dominant crop conditions (in this case maize) and optimal perennial crop conditions (in this case pigeonpea and sorghum), ultimately targeting areas where maize-based systems are likely to benefit from perennial integration. More, the data presented here are unaltered and at the remote sensing pixel level, providing a direct link to the farmer experience and offering a level of transparency (in terms of background variables) commonly masked by data aggregations and complex zoning classifications. We use geospatial technologies and techniques to (1) explore suitable (and optimal) climate conditions for pigeonpea and sorghum across Africa, (2) assess marginal maize conditions and historic agricultural productivity, and (3) identify areas where existing maize-based agricultural systems are likely to benefit from integration of sorghum or pigeonpea.

## 2 Methods

### 2.1 Fundamental climate niche: maize, pigeonpea, and sorghum

The methodology presented here consists of three major components. First, we identify the fundamental climate niches for maize, pigeonpea, and sorghum. Second, we present a range of suitability (and optimality) for each crop based on temperature and precipitation conditions. Third, we locate the intersection between marginal maize areas and the optimal pigeonpea or sorghum climate niche.

The fundamental climate niche (i.e., temperature and precipitation) used for maize, pigeonpea, and sorghum is based on a literature review of tested crop performance (Table 1). Pigeonpea is tolerant to variable rainfall, from very low to high amounts ranging from 350 to 2000 mm during a growing season (Kimani, 2000, Valenzuela and Smith, 2002, Wood and Moriniere, 2013; Houérou, n.d.), and produces grain even during dry spells, unlike other legumes (Okiror, 1986). The crop prefers temperatures ranging between 20.0 °C and 35.5 °C (Omanga et al., 1996, Carberry et al., 2001, Silim and Omanga, 2001, Sardana et al., 2010, Wood and Moriniere, 2013). Suitable growing season conditions for sorghum consist of rainfall between 150 and 950 mm (Chipanshi et al., 2003, Du Plessis, 2008, Mishra et al., 2008, Wood and Moriniere, 2013) and temperatures between 20.0 °C and 35.0 °C (Du Plessis, 2008, Wood and Moriniere, 2013; FAO, n.d.-a).

**Table 1:** Optimal temperature and precipitation ranges for maize, pigeonpea, and sorghum.

**Crop**	**Temperature (°C)**	**Precipitation (mm)**
Maize*	23.8–32.2	750–1217
Pigeonpea**	22.7–30.9	544–1263
Sorghum***	22.1–33.7	317–833

**Fig. 1:**
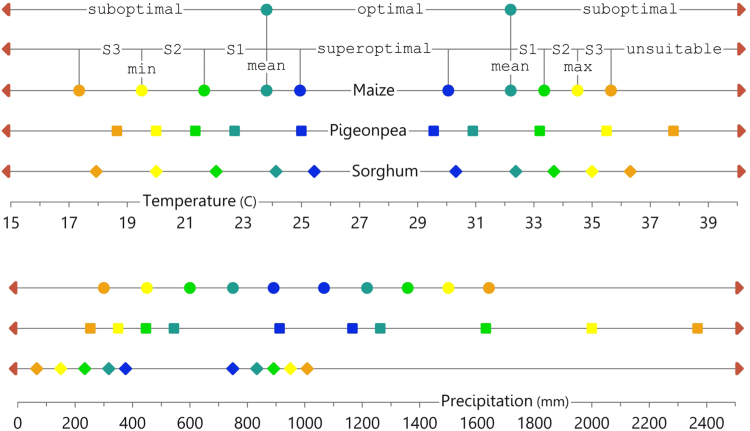
Diagram of the maize, pigeonpea, and sorghum temperature and precipitation suitability classification thresholds. Classifications include optimal and suboptimal, which were further subcategorized into a range of suitability: superoptimal, optimal, suitable 1 (S1), suitable 2 (S2), suitable 3 (S3), and unsuitable. Maize, circle; pigeonpea, square; sorghum, diamond. Suitability classification color scheme used in Fig. 3, Fig. 4. (For interpretation of the references to color in this figure legend, the reader is referred to the web version of this article).

For the optimal range, we calculated a mean low-value and mean high-value from all optimal temperature and precipitation ranges collected in our review. From the values presented in Table 1, there are two basic categories, optimal and suboptimal, which were further subcategorized into a range of suitability: superoptimal, optimal, suitable level 1 (S1), suitable level 2 (S2), suitable level 3 (S3), and unsuitable (Fig. 1). We used equal intervals from the mean low-value and high-value outward, depending on the range between the minimum or maximum value, to designate thresholds for levels of suitability. We extrapolated one step inward (toward superoptimal) and one step outward (toward unsuitable) in order to highlight an additional level of spatial variability. The same protocol was applied for classifying precipitation. The categories were constructed in this manner so that precipitation and temperature tolerance (or intolerance) would be considered when moving outward from optimal.

### 2.2 Spatial distribution of climatic variables

The spatial distribution of climate variability was acquired using two remotely-sensed NASA products. Temperature data were gathered from NASA MODIS Land Surface Temperature (LST—MYD11B3) (NASA LP DAAC, 2015a) and precipitation from NASA/JAXA Tropical Rainfall Measuring Mission (TRMM—3B43) (NASA/JAXA TRMM, 2016). Mean annual temperature and accumulated annual rainfall values were calculated between 2003 and 2014 and a conditional statement applied to classify the ranges of suitability as shown in Table 1. In order to generate suitability maps that consider both temperature and precipitation, the individual suitability maps of temperature and precipitation were overlain and reclassified so that each pixel was represented by the most unsuitable factor.

Marginal agricultural lands are those places with suboptimal temperature and precipitation conditions for the dominant crop (e.g., maize), as well as places with chronically low production levels and/or high interannual variability in production (Peter et al., in preparation). Marginal maize agricultural land is identified here using suboptimal temperature and precipitation ranges for maize (Table 1, Fig. 1), in combination with an interannual variability model that identifies areas where agricultural production is historically low and/or highly variable (Peter et al., in preparation). The model uses time-series Net Primary Productivity (NPP—MOD17A3) (NASA LP DAAC, 2015b) to categorize individual pixels based on regional productivity trends, highlighting three levels of production and two types of interannual variability. Six results are returned: Low-Stable (LS), Low-Variable (LV), Medium-Stable (MS), Medium-Variable (MV), High-Stable (HS), High-Variable (HV) (Peter et al., in preparation). From these classifications, we considered areas that are historically low and/or variable (LS and LV) to be marginal, as well as areas that are historically medium and variable (MV). Combining suboptimal temperature and precipitation conditions with marginal historic productivity reveals the spatial organization of various combinations of marginality and suboptimality, and serves to disentangle possible drivers of underproduction (Fig. 4). In order to delineate areas by agricultural land, we used a global cropland percentage map developed by Fritz et al. (2015).

Areas likely to receive maximum benefit from perennial deployment exist at the intersection between marginal dominant crop conditions (i.e., maize) and the optimal climate niche for perennial grains (i.e., pigeonpea and/or sorghum) (Peter et al., 2017). We identify this intersection by overlaying optimal perennial pigeonpea or sorghum conditions with the marginal maize areas identified above (Fig. 4). These are areas where perennial properties may serve to improve existing maize-based agricultural systems.

## 3 Results

### 3.1 Spatial organization of crop suitability

**Fig. 2:**
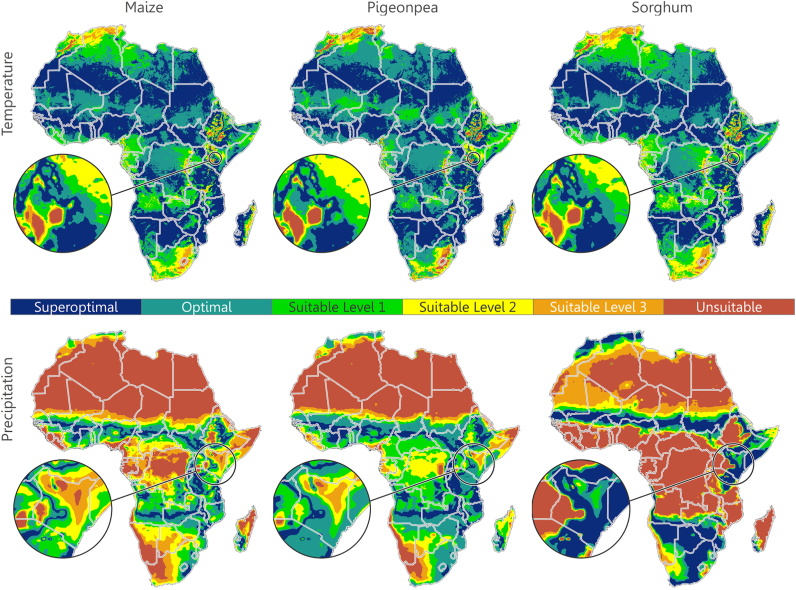
Temperature and precipitation suitability for maize, pigeonpea, and sorghum. Kenya is highlighted in order to show local spatial variability. Color scheme from Fig. 1. (For interpretation of the references to color in this figure legend, the reader is referred to the web version of this article).

Suitable temperature and precipitation conditions for maize, pigeonpea, and sorghum are spatially organized across Africa, following well-established agroecological zones (e.g., IIASA/FAO, 2012) (Fig. 2). Marked temperature suitability differences exist in the Southern and Central regions of the continent. Suitable precipitation ranges vary significantly by crop, and clear regional delineations are revealed when mapped. In terms of precipitation, sorghum, for example, is highly suitable in Southern Africa, Northern Africa (i.e., Northern Algeria, Morocco, and Tunisia), parts of Eastern Africa (i.e., Kenya and Tanzania), the Horn of Africa (i.e., Somalia, Ethiopia, Djibouti, and Eritrea), and a horizontal stretch along the Sahel. Precipitation in these areas is uniquely suitable for sorghum and is largely suboptimal or unfavorable for pigeonpea and maize. Pigeonpea has a breadth of rainfall suitability that covers much of Africa south of the Sahara. Unlike sorghum and maize, pigeonpea suitability, particularly in terms of precipitation, covers a large land area in the Central region of Africa (i.e., the Democratic republic of Congo and adjacent countries).

**Fig. 3:**
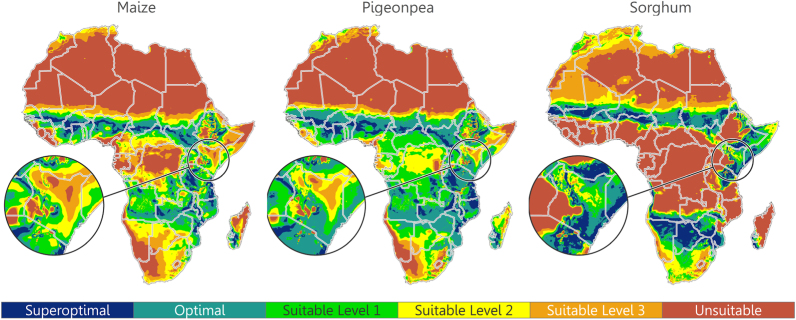
Combined suitability of temperature and precipitation suitability for maize, pigeonpea, and sorghum. Kenya is highlighted in order to show local spatial variability. Color scheme from Fig. 1. (For interpretation of the references to color in this figure legend, the reader is referred to the web version of this article).

Since suitable temperature ranges are similar among the crops under study, precipitation is the primary determinant of continental-scale suitability; however, temperature does reveal its importance when combined with precipitation, particularly at fine spatial scales (Fig. 3). The most notable distinction is that temperature restricts the suitability of sorghum in the Southern tip of Africa, as well as the North western portion of the continent (i.e., Northern Algeria, Morocco, and Tunisia); maize and pigeonpea are affected in these areas, as well.

Overall, we found that maize production is optimal on approximately 28.1% of agricultural land across Africa, suitable on 59.3%, and unsuitable on 12.6% (Fig. 3, Table 2). Pigeonpea is optimal on 44.7% of agricultural land across Africa, suitable on 49.6%, and unsuitable on 5.7%. Sorghum is optimal on 24.3% of agricultural land across Africa, suitable on 26.6%, and unsuitable on 49.1%. Sorghum is the least suitable in terms of land area; however, conditions are optimal for its cultivation where maize and pigeonpea are not. Optimal pigeonpea conditions occupy the greatest land area, and optimal conditions for maize are surprisingly low given the extent to which it is cultivated.

**Table 2:** Agricultural land area proportions for each category. Global cropland percentage map by Fritz et al. (2015) used to delineate areas by agriculture.

	**Optimal**		**Suboptimal**			
**Crop**	**Superoptimal**	**Optimal**	**Suitable Level 1**	**Suitable Level 2**	**Suitable Level 3**	**Unsuitable**
**Maize**	8.1%	20.0%	21.9%	22.0%	15.4%	12.6%
**Pigeonpea**	11.3%	33.4%	26.4%	15.5%	7.7%	5.7%
**Sorghum**	12.6%	11.7%	8.0%	9.5%	9.1%	49.1%

### 3.2 Scaling perennial innovations for maximum benefit

**Fig. 4:**
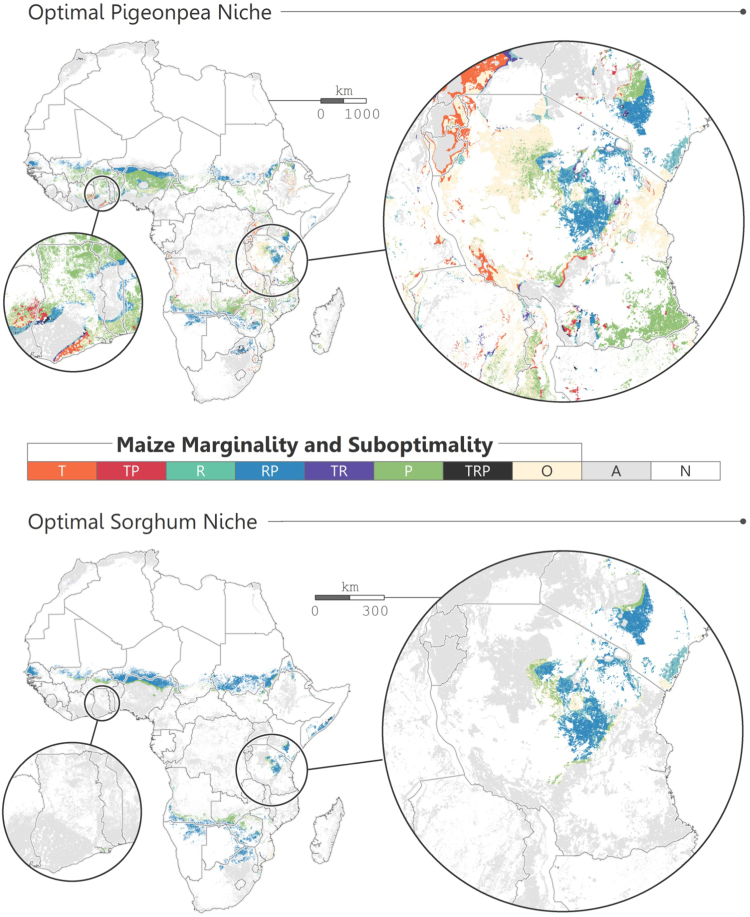
Intersection of marginal maize and optimal pigeonpea or sorghum. Areas presented in color exhibit optimal temperature and precipitation conditions for pigeonpea or sorghum. Suboptimal temperature and precipitation legend classifications are associated with the fundamental niche for maize: T = suboptimal temperature; TP = suboptimal temperature and marginal productivity; R = suboptimal rainfall; RP = suboptimal rainfall and marginal productivity; TR = suboptimal temperature and suboptimal rainfall; P = marginal productivity; TRP = suboptimal temperature, suboptimal rainfall, and marginal productivity; O = optimal; A = other agriculture (i.e., suboptimal for pigeonpea or sorghum); N = non-agriculture. Global cropland percentage map by Fritz et al. (2015) used to delineate areas by agriculture. (For interpretation of the references to color in this figure legend, the reader is referred to the web version of this article).

Within the optimal pigeonpea niche, 24.9% exhibits marginal agricultural productivity and suboptimal rainfall conditions for maize, 41.6% exhibits sole marginal agricultural productivity, and 20.9% exhibits optimal conditions for maize (Fig. 4, Table 3). Within the optimal sorghum niche, 71.4% exhibits marginal agricultural productivity and suboptimal rainfall conditions for maize, 18.2% exhibits sole marginal agricultural productivity, and only 4.6% exhibits optimal conditions for maize. Areas that exhibit sole marginal agricultural productivity are areas where marginality may be driven by social or other biogeographic factors (e.g., soil quality and land management practices). In areas where maize conditions are suboptimal and/or agricultural productivity is marginal, and pigeonpea or sorghum climate conditions are optimal, integration of these crops is likely to benefit existing maize-based agricultural systems.

**Table 3:** Agricultural land area proportions for each category. Proportions presented only within the optimal pigeonpea or sorghum niche; other agricultural land not included. Global cropland percentage map by Fritz et al. (2015) used to delineate areas by agriculture.

**Crop**	**T**	**TP**	**R**	**RP**	**TR**	**P**	**TRP**	**O**
**Pigeonpea**	4.3%	1.2%	5.2%	24.9%	1.0%	41.6%	1.0%	20.9%
**Sorghum**	< 0.1%	< 0.1%	5.4%	71.4%	< 0.1%	18.2%	0.3%	4.6%

## 4 Discussion and conclusions

### 4.1 Promoting a sustainable future

Historically, most smallholder farmers in Africa have depended on intensively managed maize systems as a cash crop and staple food (Byerlee and Heisey, 1996); however, taking a closer look reveals frequent and continual cultivation of, and dependence upon, pigeonpea and/or sorghum for food security (Rufino et al., 2013). Many African households are familiar with cultivation needs of perennial grains through generational knowledge (Rufino et al., 2013, Rogé et al., 2016), yet there is little academic attention given to perennial management practices (e.g., ratooning) of perennial grains (Kane et al., 2016). In Africa, there is potential to revitalize perennial crops such as pigeonpea and sorghum through innovative practices that offer multiple benefits (e.g., ecosystem improvements, crop water-use efficiency, and improved cultivars that enhance nutrition) (DeVries and Toenniessen, 2001, Cox et al., 2006, DeHaan et al., 2007).

Perennial grains offer benefits that encompass rural and marginal environment smallholder farming contexts, and should be promoted in locations where labor constraints reduce agricultural system efficiency, or where topography and farming practices increase erosion or depletion of soil organic matter. Integration of perennial pigeonpea or sorghum into maize-based farming systems shows wide spatial applicability across Africa. Though the optimal land area for sorghum cultivation appears of moderate size, low rainfall requirements permit it to fill a spatial niche that pigeonpea does not. This regional delineation of crop suitability between sorghum and pigeonpea is particularly evident along a horizontal stretch of the Sahel (Fig. 4). This is a marginal area for which there are few suitable crop options, and sorghum is often overlooked in a maize-centric agricultural development agenda (Hellin et al., 2012). Overall, conditions are optimal for pigeonpea cultivation on approximately 45% of agricultural land across Africa, and optimal for sorghum on 24% (Fig. 4). Between the two crops, approximately 53% of African agricultural lands are likely to benefit from their integration. There are certainly other perennials that may be promoted in Africa (e.g., rice), and there is considerable potential to improve maize-based farming systems through integration of semi- and fully perennial crops that provide multiple ecosystem services (Glover et al., 2010).

Pigeonpea diversification offers unique environmental benefits, particularly in locations where soil fertility decline is limiting crop yields. The ability of pigeonpea to biologically fix nitrogen has been proven to function in a smallholder farm context across a range of soil types, rehabilitating soils through input of 70–100 kg of nitrogen per hectare annually (Myaka et al., 2006). Given the overlap between the pigeonpea and maize niche across large swaths of agricultural lands, this could potentially address a substantial portion of the nitrogen deficit that is a primary yield limiting factor on African smallholder farms (Vanlauwe et al., 2011). Sorghum is more drought-resistant than maize, yet it has been replaced by maize in areas such as Northern Malawi (Bezner Kerr, 2014). Where it is still grown, farmers in Malawi ratoon by cutting the stem to extend production for more than one season, and have reported that ratooning also saves labor and seeds (Rogé et.al, 2016). Smallholder farmers typically cultivate sorghum in mixed cropping systems where the spatial arrangement of crops plays a major role. In Malawi, for example, farmers have reported growing sorghum on soils that are marginal for maize (Rogé et al., 2016). In North-Central Nigeria, sorghum has been reported to be intercropped in combinations with millet, groundnut, and/or cowpea (Okigbo and Greenland, 1976). Due to current global climate impacts on crops, there have been partial efforts for breeding of sorghum (ICRISAT, n.d.), and research shows that breeding sorghum can produce varieties adaptable to climate variability and pests (Haussmann et al., 2012).

Despite the legacy cultivation of maize across much of Africa, and recent expansion into new areas of West and North Africa (Blackie and Dixon, 2016), there are large areas that are suboptimal environments for maize growth. The decision to cultivate maize in these areas reflects socioeconomic factors such as market demand and farmer preference, and may also be influenced by the superior calories per unit input that maize offers (Snapp and Silim, 2002). Interestingly, our analysis shows that the Horn of Africa is largely unsuitable for maize production and optimal for sorghum, yet maize is being widely promoted. One study found that one-third of farmers ratoon sorghum for perennial production in lowland areas of Ethiopia (Mekbib, 2009), yet surprisingly little research focuses on this drought-adaptation option, even in the center of origin for the crop. Overall, our findings highlight the role that both sorghum and pigeonpea could play in improving food security and resilience across marginal swaths of Africa. This is further evidence that the crop has been understudied, particularly for novel perennial genotypes and historically important ratooned types (Paterson et al., 2014).

### 4.2 Conclusions

Sustainable land management is at the forefront of agronomic research, and nature-based solutions are emerging as long-term subsistence strategies. Following nature-based models, sustainable agricultural solutions for smallholder farms are innovations inspired by nature. Though many nature-based solution initiatives in the European context have been urban-centric (Nesshöver et al., 2016), the core sustainable intensification concepts have broad potential. In rural Africa, where land availability and fertilizer access are often limited, smallholder farming systems may benefit from the sustainable cropping methods and synergistic crop choices that nature-based solutions promote. As arable, fertile land continues to diminish and population growth continues to drive production demand, proactive strategies for increased and sustainable production need to be considered. There are certainly other perennials that may be promoted in Africa, and the potential to improve maize-based farming systems through perennial integration is substantial, and underappreciated. Though we present the spatial suitability of maize, pigeonpea, and sorghum in Africa, the heuristic proposed is generalizable and not limited to any particular set of crops or region. We recommend that policy- and decision-makers utilize information and techniques such as these to intelligently target areas that may benefit from perennial integration. As pigeonpea, sorghum, and other perennials are reintroduced to parts of Africa where perennial production has been neglected in preference for maximizing maize yields, it is crucial to promote cultivation of these environmental options in areas where climate conditions are conducive to prosperous and synergistic growth.
